# Safety and feasibility of intravenous administration of a single dose of allogenic-Muse cells to treat human cervical traumatic spinal cord injury: a clinical trial

**DOI:** 10.1186/s13287-024-03842-w

**Published:** 2024-08-13

**Authors:** Masao Koda, Shiro Imagama, Hiroaki Nakashima, Sadayuki Ito, Naoki Segi, Jun Ouchida, Kota Suda, Satoko Harmon Matsumoto, Miki Komatsu, Toshiki Endo, Shinsuke Suzuki, Satoshi Inami, Haruki Ueda, Masayuki Miyagi, Gen Inoue, Masashi Takaso, Keiji Nagata, Hiroshi Yamada, Naosuke Kamei, Toshio Nakamae, Hidenori Suzuki, Norihiro Nishida, Masahiro Funaba, Gentaro Kumagai, Takeo Furuya, Yu Yamato, Toru Funayama, Hiroshi Takahashi, Masashi Yamazaki

**Affiliations:** 1 Department of Orthopedic Surgery, Faculty of Medicine, University of Tsukuba, Tsukuba, Japan; 2 Department of Orthopaedics/Rheumatology/Hand Surgery, Nagoya University Graduate School of Medicine, Nagoya, Japan; 3 Hokkaido Spinal Cord Injury Center, Bibai, Japan; 4 Department of Neurosurgery, Sendai Medical Center, Sendai, Japan; 5 Division of Neurosurgery, Tohoku Medical and Pharmaceutical University, Sendai, Japan; 6 Department of Orthopedic Surgery, Dokkyo Medical University, Mibu, Japan; 7 Department of Orthopedic Surgery, Kitasato University School of Medicine, Sagamihara, Japan; 8 Orthopedic Surgery, Wakayama Medical University, Wakayama, Japan; 9 Department of Orthopaedic Surgery, Graduate School of Biomedical and Health Sciences, Hiroshima University, Hiroshima, Japan; 10 Department of Orthopedic Surgery, Yamaguchi University School of Medicine, Yamaguchi, Japan; 11 Department of Orthopaedic Surgery, Hirosaki University Graduate School of Medicine, Hirosaki, Japan; 12 Department of Orthopedic Surgery, Graduate School of Medicine, Chiba University, Chiba, Japan; 13 Department of Orthopedic Surgery, Hamamatsu University School of Medicine, Hamamatsu, Japan

**Keywords:** Spinal cord injury, Regenerative therapy, Muse cell, Clinical trial

## Abstract

**Introduction:**

Spinal cord injury (SCI) is a devastating injury and remains one of the largest medical and social burdens because of its intractable nature. According to the recent advances in stem cell biology, the possibility of spinal cord regeneration and functional restoration has been suggested by introducing appropriate stem cells. Multilineage-differentiating stress enduring (Muse) cells are a type of nontumorigenic endogenous reparative stem cell. The positive results of Muse cell transplantation for SCI was shown previously. As a first step for clinical application in human SCI, we conducted a clinical trial aiming to confirm the safety and feasibility of intravenously injected donor-Muse cells.

**Methods:**

The study design of the current trial was a prospective, multicenter, nonrandomized, nonblinded, single-arm study. The clinical trial registration number was JRCT1080224764. Patients with a cervical SCI with a neurological level of injury C4 to C7 with the severity of modified Frankel classification B1 and B2 were included. A primary endpoint was set for safety and feasibility. Our protocol was approved by the PMDA, and the trial was funded by the Life Science Institute, Tokyo, Japan. The present clinical trial recruited 10 participants (8 males and 2 females) with an average age of 49.3 ± 21.2 years old. All 10 participants received a single dose of allogenic CL2020 (a total of 15 × 10^6^ cells, 2.1–2.7 × 10^5^ cells/kg of body weight), which is a Muse cell-based product produced from human mesenchymal stem cells, by an intravenous drip.

**Results:**

There were two reported severe adverse events, both of which were determined to have no causal relationship with Muse cell treatment. The change in the ISNCSCI motor score, the activity of daily living and quality of life scores showed statistically significant improvements compared to those data at the time of CL2020 administration.

**Conclusion:**

In the present trial, no safety concerns were identified, and Muse cell product transplantation demonstrated good tolerability. Future clinical trials with appropriate study designs incorporating a control arm will clarify the definitive efficacy of single-dose allogenic Muse cell treatment with intravenous administration to treat SCI.

*Trial registration*: jRCT, JRCT1080224764. Registered 03 July 2019, https://jrct.niph.go.jp/latest-detail/jRCT1080224764.

## Introduction

Spinal cord injury (SCI) is a devastating injury caused mainly by fracture, dislocation, and torsion of the spinal column, leading to motor/sensory/bladder palsies and long-lasting sequelae. Its annual incidence ranges from 8 to 50 per million worldwide. Because of its intractable nature, SCI remains one of the largest medical and social burdens [1].

From the early twentieth century, an injured adult mammalian central nervous system, including the spinal cord, was considered incapable of regeneration. Treatments for SCI have been confined to several options: spinal surgeries for decompression and stabilization to initiate early rehabilitation, management for complications such as pneumonia, pulmonary embolism, decubitus ulcer, and urinary tract infection, and rehabilitation that applies to remaining function for activities of daily living [2].

According to the recent advances in neuroscience and stem cell biology, the possibility of spinal cord regeneration and functional restoration has been suggested by altering the nonpermissive milieu for regeneration to a permissive microenvironment by introducing appropriate stem cells. Various types of stem cells have been reported to be effective in animal studies, and some of them have been advanced to clinical trials [3].

Multilineage-differentiating stress enduring (Muse) cells are a type of nontumorigenic endogenous reparative stem cell with dual pluripotent-like and macrophage/monocyte-like phenotypes that reside in the bone marrow as pluripotent surface marker stage-specific embryonic antigen (SSEA)-3-positive cells at a ratio of approximately 0.03% [4]. They are constantly mobilized to the peripheral blood and are distributed to nearly every organ to contribute to daily minute repair. They are also contained in cultured mesenchymal stem cells (MSCs) and fibroblasts at several percent. Tissue repair is unique: Muse cells phagocytose damaged/apoptotic cells and recycle factors, including transcription factors that are active in the phagocytosed cells, and then differentiate into the same cell type as damaged/apoptotic cells to replace them [5]. The process of differentiation proceeds on a daily basis. Due to the expression of sphingosine-1-phosphate (S1P) receptor 1, they sense S1P produced by damaged/apoptotic cells and migrate to the damaged sites, like macrophages/monocytes. Notably, donor-derived Muse cells can escape immune rejection and survive in the host tissue for an extended time due to specific immune privilege represented by the expression of HLA-G, one of the key factors for immunotolerance in the placenta. Therefore, Muse cells do not require surgery to deliver to damaged tissue; instead, intravenous injection (iv) is the main method for administration. They do not require gene introduction or cytokine induction to induce differentiation, and furthermore, HLA-matching tests or immunosuppressant treatment are unnecessary for the administration of these donor cells [6, 7]. The safety and efficacy of systemically administered donor-Muse cells have been reported in clinical trials for acute myocardial infarction [8] and epidermolysis bullosa [9].

In preclinical studies, human Muse cells selectively homed to the infarct site 1 day after iv in a lacuna stroke model [10] and to the cervical and lumbar spinal cord 7 days after iv in an ALS model [11] and were shown to differentiate into neuronal and glial cells that were incorporated into authentic neuronal circuits and delivered locomotive and sensory functions. In a rat 9th thoracic spine level contusion model of SCI, clinical-grade human Muse cells intravenously injected at 1 day integrated into the injured site, differentiated into MAP-2-positive cells, promoted the preservation of serotonin-positive nerve fibers and delivered statistically significant recovery in the BBB locomotor scale compared with the vehicle group for up to 56 days (*p* < 0.001) [12]. The positive results obtained in these studies have generated significant enthusiasm for the potential of Muse cell treatment in human SCI.

As a first step for clinical application in human SCI, we conducted a phase 1/2a clinical trial, aiming to confirm the safety/feasibility and preliminary efficacy of intravenously injected donor-Muse cells without HLA-match testing or immunosuppressant treatment for patients with traumatic SCI.

## Patients and methods

### Study design

The study design of the current trial was a prospective, multicenter, nonrandomized, nonblinded, single-arm study. The clinical trial registration number is JRCT1080224764. The current trial included patients with a cervical SCI with a neurological level of injury C4 to C7 with the severity of modified Frankel classification B1 and B2 (Ref), which indicates complete motor paralysis and remaining touch sense in the perineal area (B1) or anywhere in the patient’s body (B2) without pinprick sensation. If the pinprick sensation remained, the patients were classified into modified Frankel B3 and excluded from the trial. The reason why we included patients with modified Frankel B1/B2 was that there is large discrepancy of recovery potential between modified Frankel B1/B2 and B3 patients. Fukuda and Ueta reported that approximately 80% of patients with B3 palsy recovered to modified Frankel D, which means ambulatory status. By contrast, only 20% of those with B1/B2 recovered to modified Frankel D [13]. Thus, we chose modified Frankel B1/B2 as candidates for the present trial to distinguish Muse cells’ therapeutic potential from natural recovery.

The possible candidates were recruited within 2 weeks after injury. Patients were reassessed for neurological status at 3 weeks postinjury, and those who maintained modified Frankel classification B1/B2 were enrolled. The detailed inclusion and exclusion criteria are shown in Table 1.

**Table 1:** Inclusion and exclusion criteria

[*Inclusion criteria*]
(1) Patients within 14 days ± 2 days (from 12 to 16 days) after spinal cord injury
(2) Patients aged 16 years or older but younger than 75 years at the time of consent (regardless of sex)
(3) Patients who provided written consent themselves. In the case of patients who were minors, consent was obtained from the legal representative in writing, and whenever possible, consent was also obtained from the patient themselves through a consent document. In cases where the patient’s condition made it difficult for them to sign, a family member or equivalent partner (e.g., common-law spouse) could sign as a witness. However, once a patient was able to sign on their own, written consent was obtained from the patient themselves
(4) Patients with a modified Frankel classification of B1 or B2 at the start of screening
(5) Patients who can receive CL2020 administration within 21 days ± 3 days (from 18 to 24 days) after spinal cord injury. (6) Patients with the primary site of spinal cord injury in the cervical spine (C4 to C7)
(6) Patients who can visit for the specified evaluation periods
[Exclusion Criteria]
(1) Patients with Japan Coma Scale III-200 or III-300 consciousness disorders at the time of obtaining consent
(2) Patients with severe respiratory disorders
(3) Patients with systolic blood pressure exceeding 140 mmHg or diastolic blood pressure exceeding 90 mmHg despite antihypertensive treatment
(4) Patients with a current or past history of malignant tumors (patients who have achieved complete remission for at least 5 years are eligible for enrollment)
(5) Patients with concurrent neurological disorders, cerebrovascular disorders, or musculoskeletal disorders that may affect neurological assessment
(6) Patients who are expected to experience a rapid deterioration of symptoms related to spinal cord injury during the trial period
(7) Patients who are unable to start rehabilitation early due to complications or other reasons
(8) Patients with severe dementia or mental disorders
(9) Patients with severe spinal cord or spinal disorders (including osteoporosis, spinal tumors, spinal vascular malformations, or syringomyelia)
(10) Patients who require medical treatment for positive HBs antigen, HCV antibody, HIV antibody, or syphilis test
(11) Patients with severe connective tissue diseases and related disorders
(12) Patients with severe diabetes mellitus (HbA_1c_ ≥ 10%), regardless of treatment
(13) Patients with severe systemic conditions, such as severe infections (sepsis, etc.)
(14) Patients with severe complications or conditions deemed inappropriate by the responsible/investigating physician
(15) Patients receiving systemic administration of immunosuppressive agents
(16) Female patients who are pregnant or breastfeeding or potentially pregnant female patients
(17) Male patients who cannot agree to contraception based on the guidance of the responsible/investigating physician from consent acquisition to observation completion or female patients of childbearing potential who cannot agree to contraception
(18) Patients who participated in another clinical trial within the past 3 months before obtaining consent or received administration or use of regenerative medicine products within the past 1 year
(19) Patients with a history of hypersensitivity to aminoglycoside antibiotics, including kanamycin
(20) Patients with a history of hypersensitivity to human serum albumin products or xenogeneic proteins (bovine and porcine)
(21) Patients deemed inappropriate for participation in the clinical trial by the responsible/investigating physician for general safety reasons

Potential participants were screened by Inclusion and exclusion criteria at the time of obtaining consent. *Rationale for inclusion criteria.* (1), (5), (6) These criteria were set to select severely injured spinal cord patients appropriate for evaluating the efficacy of CL2020. (2) Based on the age distribution of registered injured patients in the database of the Kibi Plateau Medical Rehabilitation Center, patients < 16 years of age are extremely rare. Therefore, the lower age limit was set at 16 years. Although 16 years is still considered a minor, they are capable of understanding the nature of the clinical trial. Hence, the minimum age requirement was set at 16 years. Patients over 75 years of age have a high likelihood of poor functional recovery and are not suitable for evaluating efficacy. Therefore, the upper age limit was set at < 75 years. (3) This criterion was set to protect human rights in accordance with the Helsinki Declaration. In addition, the inclusion of minors was allowed, so consent was obtained from the legal representatives and ascent from the patients themselves. (4) Patients with a modified Frankel classification of B1 or B2 were selected because they have limited prospects for significant recovery with existing treatments. (7) This criterion was set to ensure that the evaluation of the investigational product could be appropriately confirmed at the participating medical institutions. *Rationale for exclusion criteria.* (1)–(15) These criteria were set to exclude patients who may impact the safety and efficacy evaluation of CL2020. (16), (17) CL2020 has not been studied for safety regarding administration during pregnancy, to the fetus, or during breastfeeding; thus, these exclusion criteria were set. (18) This criterion was set to exclude the influence of other investigational drugs or investigational products for which the evaluation has not been confirmed. (19) Kanamycin is used in the manufacturing process, and this criterion was set to ensure the safety of the subjects. (20) This criterion was set to consider the safety of the subjects due to the use of human serum albumin products and materials derived from xenogeneic sources (bovine and porcine) in the manufacturing process. (21) This criterion was set for general safety considerations

### Muse cells

We used CL2020, which is a Muse cell-based product produced from human mesenchymal stem cells (MSCs), after exposing the cells to combination stresses (Life Science Institute) for transplantation. The Muse cells were enriched with magnetic cell sorting for SSEA-3-positive fraction from mesenchymal stem cells collected from bone marrow aspiration of Oriental Asian volunteers. Cell characterization was performed as followings. Therapeutic potential was evaluated by administration to spinal cord injured rats. As safety pharmacology studies, the effects on the respiratory system in rats and the cardiovascular system in monkeys were examined. There was no adverse events. In pharmacokinetic studies, radioisotope-labelled Muse cells were administered intravenously to normal rats, and quantitative whole-body autoradiography was performed. Toxicity studies evaluated general toxicity and carcinogenicity of Muse cell products. There was no toxicity nor carcinogenicity.

The participants were administered 15 × 10^6^ Muse cells (2.1–2.7 × 10^5^ cells/kg of body weight) in normal saline via intravenous drip infusion. The dosage was determined based on the results from animal studies according to the instructions of the Japanese Pharmaceutical and Medical Device Agency (PMDA) for consolidation of product labeling.

### Endpoints

A primary endpoint was set for safety/feasibility and preliminary efficacy. To evaluate safety, all adverse events, which refer to any symptom or disease signs in a participant after informed consent with or without a causal relationship with CL2020 treatment, were collected, and then adverse event-related terminologies were coded by the investigators according to the ICH International Medical Dictionary for Regulatory Activities Japanese version (MedDRA/J) for up to 52 weeks after administration. Anaphylaxis and pulmonary embolism were assumed to be adverse events related to the treatment at the acute phase that should be given full attention.

To evaluate preliminary efficacy, we set improvement of modified Frankel grade as one of the primary endpoints.

The secondary endpoints were as follows. (1) Changes in the International Standards for Neurological Classification of Spinal Cord Injury 1992 version (ISNCSCI) motor score were evaluated from the screening completion to 4, 12, 28, and 52 weeks after administration. The ISNCSCI score was evaluated in each subdivision, namely, the upper extremity motor score (UEMS), lower extremity motor score (LEMS), and total motor score, and (2) changes in the ISNCSCI sensory score both of light touch and pinprick from the screening completion to 4, 12, 28, and 52 weeks after administration. (3) change in Spinal Cord Independence Measure version III (SCIM) for activities of daily living assessment, (4) change in EuroQol 5 Dimension 5 Level [EQ-5D-5 L] for quality-of-life assessment were also examined.

Our protocol was approved by the PMDA, and the trial was funded by the Life Science Institute, Tokyo, Japan.

### Statistical analyses

For the motor/sensory ISNSCI scores, SCIM score, and EQ-5D score, summary statistics and the mean value were calculated for the end of the screening, and 4, 12, 28, and 52 weeks after CL2020 administration, along with the two-sided 95% confidence interval (CI). A linear mixed-effects model was used to calculate the adjusted mean value and its two-sided 95% CI, as well as the *p* value adjusted for baseline values. The model considered the time point and baseline value as fixed effects, and the correlation structure between time points in the subjects was determined considering estimability. The Kenward–Roger method was used to calculate degrees of freedom. Similarly, an analysis was conducted for the change in scores from the end of screening to 52 weeks after CL2020 administration. All statistical analyses were performed using SAS 9.4 statistical software (SAS Institute, Cary, NC).

### Ethics

The present study was approved by the Institutional Review Board (IRB) of each institution that participated in the present trial and was conducted according to the principles of the World Medical Association (WMA) Declaration of Helsinki-Ethical Principles for Medical Research Involving Human Subjects with the amendments made in 2013: Seventh revision, 64th Meeting, Fortaleza, Brazil, with a Note of Clarification on Paragraph 29 added by the WMA General Assembly, Washington 2002; Note of Clarification on Paragraph 30 added by the WMA General Assembly, Tokyo 2004, and in accordance with the Japanese Medical Research Involving Human Subjects Act (WMO) and other guidelines, regulations and acts.

### Patient informed consent

Written informed consent was obtained from the patient and the patient’s legal representative. In cases where the trial participant could not physically sign the informed consent form because of upper extremity palsy caused by SCI, allographs provided by the patients’ representatives were allowed.

## Results

Detailed demographics of the patients are shown in Table 2. The present clinical trial recruited 10 participants (8 males and 2 females) with an average age of 49.3 ± 21.2 years old. The severity of SCI was assessed using the modified Frankel classification, with 4 cases classified as B1 and 6 cases as B2. The neurological level of injury varied: 5 cases at C4, 1 case at C5, 3 cases at C6, and 1 case at C7. The types of spinal cord injuries included dislocation fractures in 5 cases, fractures in 2 cases and 3 cases without bony abnormalities. All the participants underwent surgical decompression and/or fixation. None of the patients received steroid administration.

**Table 2:** Patient demographics

Patient demographics (n = 10)	
Male: female	8: 2
Mean age at injury (years old)	49.3 (27–67)
Height (cm)	170.28 (163.2–179.2)
Body weight (kg)	61.3 (55.5–70.0)
Body mass index (kg/m^2^)	21.2 (17.3–24.8)
Modified Frankel grade (cases)	
B1	4
B2	6
Neurological level of injury (cases)	
C4	5
C5	1
C6	3
C7	1
Bony injury (cases)	
Fracture-dislocation	5
Fracture	2
Without bony injury	3

All 10 participants received a single dose of allogenic CL2020 (15 × 10^6^ cells corresponding to 2.1 to 2.7 × 10^5^ cells/kg body weight) by intravenous drip. There were no cases in which Muse cell administration was discontinued during the procedure.

There were two reported severe adverse events. One patient showed mortality (10%) during the follow-up period, which was attributed to aspiration pneumonia. The participant developed aspiration pneumonia 60 days after CL2020 administration, and respiratory management was performed through endotracheal intubation and tracheostomy, along with antibiotic treatment. The symptoms were temporarily relieved, but the respiratory condition deteriorated again, leading to respiratory failure and death 193 days after treatment. Aspiration pneumonia related to the underlying condition, insufficient tidal volume due to thorax movement palsy caused by SCI, was determined to be the primary cause of death and was determined to have no causal relationship with Muse cell treatment.

Another patient had a urinary tract stone that needed surgical treatment. The patient was an 18-year-old man without a notable medical history who had a bladder stone on day 354 that needed endoscopic stone excision, probably because of the sequela of a neurogenic bladder and repeated urinary tract infection due to bladder palsy caused by SCI. Therefore, this event was also considered to be caused by an underlying condition unrelated to CL2020 administration.

**Fig. 1:**
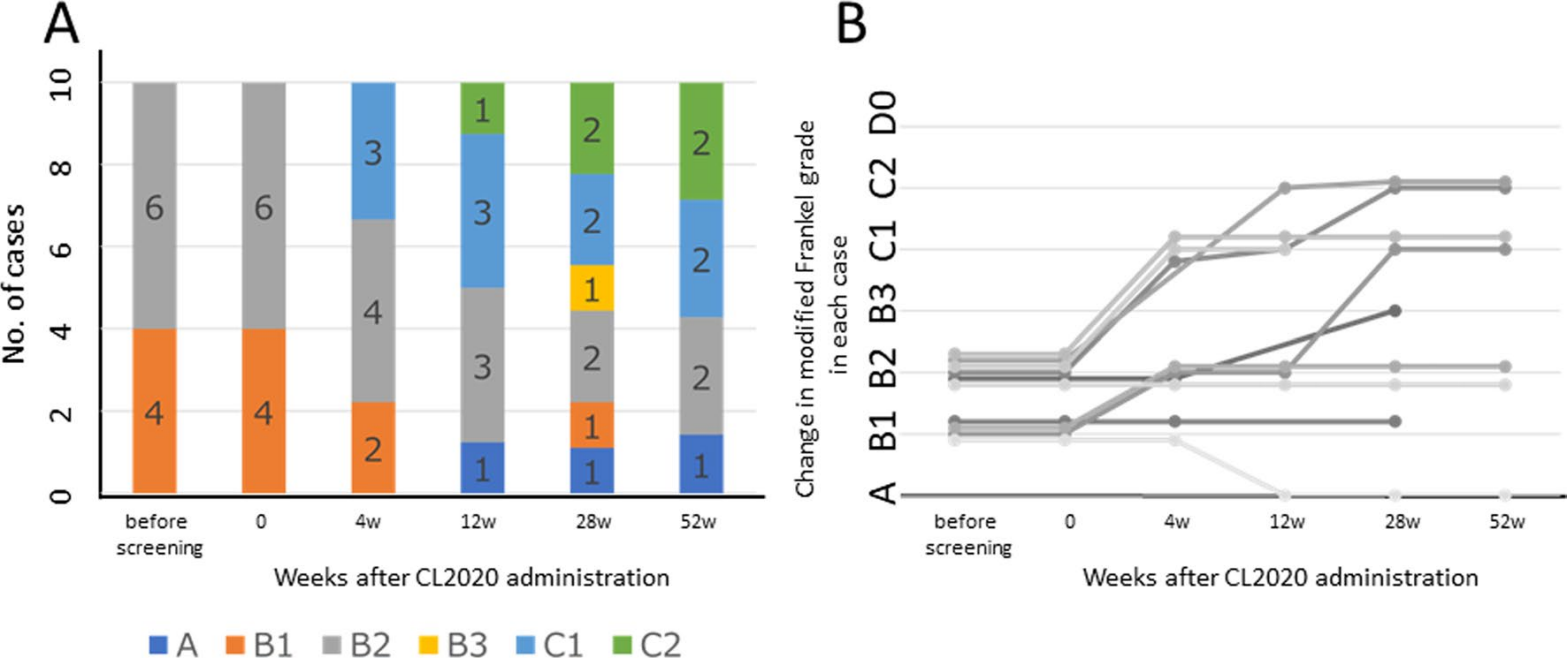
Change in modified Frankel grade. Panel** A** shows change in the number of cases of each modified Frankel grade in chronological order. Panel** B** shows change in modified Frankel grade over time in each case

The modified Frankel classification showed an improvement of at least one grade in 6 of 10 participants at 28 weeks posttreatment and in 5 of 10 participants at 52 weeks posttreatment (evaluation was incomplete after 28 weeks for one participant who died due to aspiration pneumonia) (Fig. 1A, B).

**Fig. 2:**
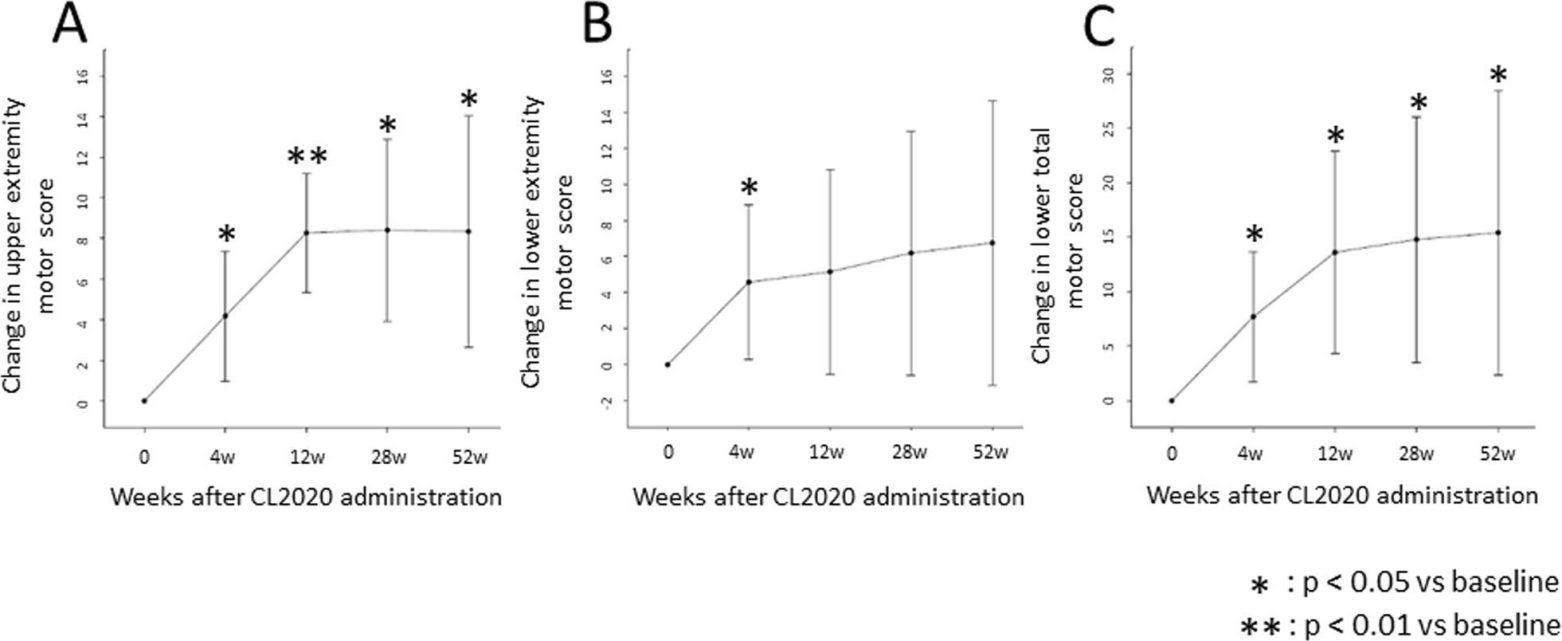
Change in ISNCSCI motor score. The change in the ISNCSCI upper extremity motor score **A** was 4.2 [1.0–7.4] (adjusted mean [95% CI]) at 4 weeks posttreatment (*p* = 0.016), 8.3 [5.3–11.2] at 12 weeks (*p* < 0.001), 8.4 [3.9–12.9] at 28 weeks (*p* = 0.002), and 8.3 [2.7–14.0] at 52 weeks (*p* = 0.009), showing statistically significant improvements at each time point compared to those data at the time of CL2020 administration. The change in the ISNCSCI lower extremity motor score **B** showed significant improvement at 4 weeks post-treatment (*p* < 0.05) but did not show statistically significant improvements after subsequent time points compared to the time of CL2020 administration. The changes in the total ISNCSCI motor score **C** were 7.7 [1.7–13.6] (*p* = 0.020), 13.6 [4.3–22.9] (*p* = 0.009), 14.8 [3.5–26.0] (*p* = 0.016), and 15.4 [2.3–28.5] (*p* = 0.026) at 4, 12, 24, and 52 weeks posttreatment, respectively, with statistically significant improvements at each time point compared to the time of CL2020 administration

The change in the ISNCSCI upper extremity motor score was 4.2 [1.0–7.4] (adjusted mean [95% CI]) at 4 weeks posttreatment (*p* = 0.016), 8.3 [5.3–11.2] at 12 weeks (*p* < 0.001), 8.4 [3.9–12.9] at 28 weeks (*p* = 0.002), and 8.3 [2.7–14.0] at 52 weeks (*p* = 0.009), showing statistically significant improvements at each time point compared to those data at the time of CL2020 administration (Fig. 2A). The change in the ISNCSCI lower extremity motor score (LEMS) showed significant improvement at 4 weeks post-treatment (*p* < 0.05) but did not show statistically significant improvements after subsequent time points compared to the time of CL2020 administration (Fig. 2B). The changes in the total ISNCSCI motor score were 7.7 [1.7–13.6] (*p* = 0.020), 13.6 [4.3–22.9] (*p* = 0.009), 14.8 [3.5–26.0] (*p* = 0.016), and 15.4 [2.3–28.5] (*p* = 0.026) at 4, 12, 24, and 52 weeks posttreatment, respectively, with statistically significant improvements at each time point compared to the time of CL2020 administration (Fig. 2C).

**Fig. 3:**
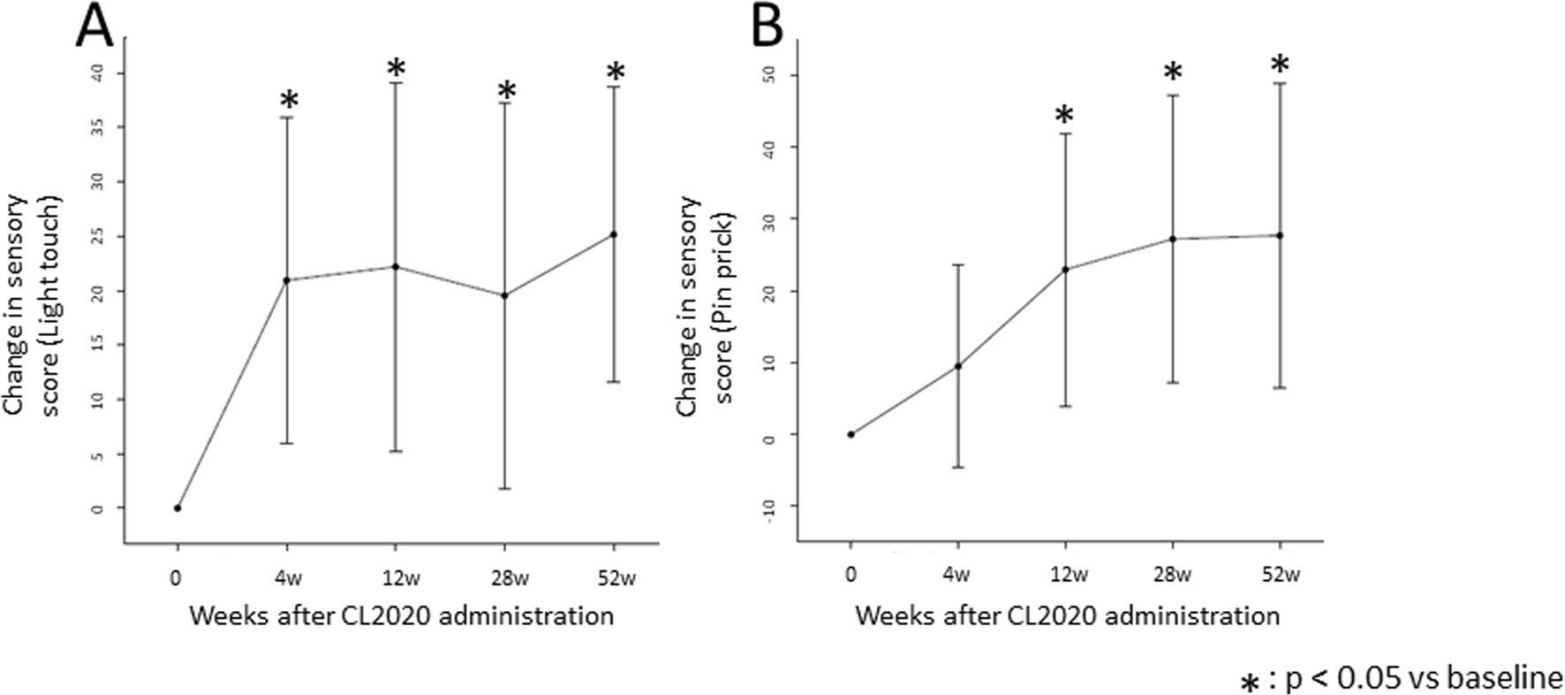
Change in ISNCSCI sensory score. Changes in ISNCSCI sensory score for both light touch (**A**) and pinprick (**B**) showed statistically significant improvements at all time points except pinprick at 4 weeks compared to the time of CL2020 administration

The change in the ISNCSCI sensory score (pinprick sensation) from the screening end was 9.5 [− 4.6 to 23.6] at 4 weeks (*p* = 0.161), 22.9 [4.0 to 41.9] at 12 weeks (*p* = 0.023), 27.2 [7.3 to 47.1] at 28 weeks (*p* = 0.014), and 27.7 [6.5 to 48.8] at 52 weeks (*p* = 0.017), with statistically significant improvements after 12 weeks compared to the time of CL2020 administration. Light touch and pinprick showed statistically significant improvements at all time points except pinprick at 4 weeks compared to the time of CL2020 administration (Fig. 3A, B).

**Fig. 4:**
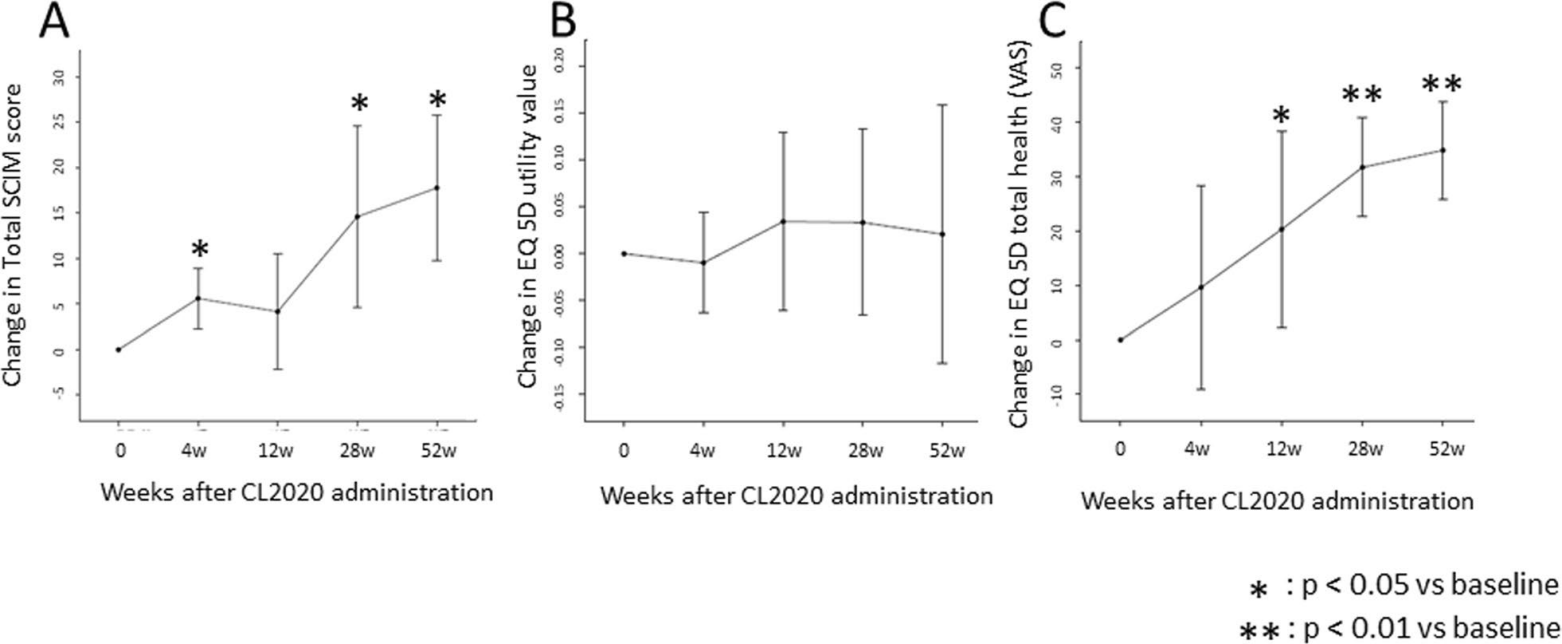
Change in activity of daily living and quality of life scores. The change in the total score of the spinal cord independence measure (SCIM) **A** was 5.6 [from 2.3 to 8.9] at 4 weeks (*p* = 0.006), 4.2 [− 2.2 to 10.5] at 12 weeks (*p* = 0.167), 14.6 [4.6 to 24.6] at 28 weeks (*p* = 0.011), and 17.8 [9.7 to 25.8] at 52 weeks (*p* = 0.002), with statistically significant improvements at all time points except 12 weeks. The change in the quality-of-life (QOL) values evaluated with the EQ-5D (**B**) did not show significant improvements at any time. By contrast, the change in the perception of current health status (**C**) showed statistically significant improvements after 12 weeks compared to the time of CL2020 administration

The change in the total score of the SCIM was 5.6 [from 2.3 to 8.9] at 4 weeks (*p* = 0.006), 4.2 [− 2.2 to 10.5] at 12 weeks (*p* = 0.167), 14.6 [4.6 to 24.6] at 28 weeks (*p* = 0.011), and 17.8 [9.7 to 25.8] at 52 weeks (*p* = 0.002), with statistically significant improvements at all time points except 12 weeks (Fig. 4A).

The change in the quality-of-life (QOL) values evaluated with the EQ-5D did not show significant improvements at any time (Fig. 4B). By contrast, the change in the perception of current health status was 9.6 [− 9.2 to 28.4] at 4 weeks (*p* = 0.268), 20.3 [2.3 to 38.3] at 12 weeks (*p* = 0.032), 31.7 [22.6 to 40.7] at 28 weeks (*p* < 0.001), and 34.8 [25.8 to 43.8] at 52 weeks (*p* < 0.001), with statistically significant improvements after 12 weeks compared to the time of CL2020 administration (Fig. 4C).

The modified Frankel classification showed an improvement of at least one grade in 6 of 10 participants at 28 weeks posttreatment and in 5 of 10 participants at 52 weeks posttreatment (evaluation was incomplete after 28 weeks for one participant who died due to aspiration pneumonia) (Fig. 1A, B).

Based on these findings, the administration of Muse cells to subacute phase cervical SCI was associated with improvements in functional impairment and QOL.

## Discussion

In this clinical trial, two severe adverse events occurred, namely, mortality and urinary tract stones. The safety committee considered those adverse events to be attributed to the underlying conditions (aspiration pneumonia, thorax hypomobility caused by motor palsy of SCI; and urinary tract stone, neurogenic bladder due to bladder palsy caused by SCI) and not directly related to CL2020 administration. Because CL2020 is a cell processing product derived from allogeneic bone marrow, there are concerns regarding the risks of anaphylaxis and complications after intravenous administration, such as cell embolism, as described in the package insert for Temcell HS, which is also an allogeneic bone marrow-derived mesenchymal stem cell product. However, no adverse events suggestive of these complications were observed in this trial. Consequently, safety concerns were not identified, and CL2020 administration demonstrated good tolerability, suggesting that a single dose of allogenic CL2020 treatment with an intravenous drip was suggested to be safe and feasible for severe human cervical SCI.

Although modified Frankel grade, motor and sensory ISNSCI scores, SCIM, and EQ5D showed significant improvement compared with those before treatment, definitive conclusions on efficacy cannot be clearly drawn because the present study was conducted by a single-arm design without setting control subjects. To date, it is impossible to compare directly with historical data because no appropriate historical control data for motor/sensory and ADL/QOL recovery are generally available. Alternatively, we compared our data with previously published data based on intravenous infusion of autologous mesenchymal stem cells (MSCs) reported by Honmou et al. [14]. As this product was autologous MSCs cultured from bone marrow aspirate that might have included several percent of Muse cells, both might fall into a similar category. However, the data cannot be compared directly between MSCs and Muse cells because there are distinct differences in cellular phenotype, inclusion criteria, and evaluation methods. Honmou et al. targeted AIS A/B/C patients. Our inclusion criteria, Modified Frankel B1/B2, are defined as complete motor palsy with touch sensation but without pinprick sensation, possibly corresponding to AIS A (complete motor and sensory palsies but potentially including modified Frankel B1 because it is difficult to diagnose when it shows remaining touch sense only in the perineal area) and B (potentially including modified Frankel B3). Therefore, we extracted data from patients with AIS A and B from the report by Honmou et al., which included 8 patients with AIS A/B of 13 patients. Three of 8 patients with AIS A/B showed 1-level AIS improvement (37.5%), 4 showed 2-level AIS improvement (50%), and one patient showed no change (12.5%). In the present study, 4 of 10 patients showed modified Frankel classification B1/B2 to C improvement (40%), another 4 showed no change (40%), and the remaining patient showed deterioration from B1 to A (10%). These results suggested at least no inferiority of Muse cell treatment to the treatment in the study by Honmou et al. Direct comparison between MSC and Muse cell transplantation could be performed by setting the MSC group as a control. However, MSCs were tentatively approved by the Japanese government, and postmarket study is now underway. Thus, direct comparison cannot currently be permitted.

Fukuda and Ueta from Sogo Sekison Center (Japanese Spinal Injury Center, Izuka, Japan) reported the natural recovery course after cervical SCI using a modified Frankel classification [13]. They showed a clear discrepancy in recovery rate between patients with B1/B2 and those with B3. Approximately 80% of patients with B3 palsy recovered to modified Frankel D, which means ambulatory status. By contrast, only 20% of those with B1/B2 recovered to modified Frankel D. Although the data by Fukuda and Ueta are informative, they cannot be directly compared with our data because the time of the initial evaluation was largely different: being at admission (1.7 days after the injury on average) in their study and 3 weeks after injury in our study. It is more suitable to compare Muse cell data and possible control with the precise time point, namely, 3 weeks post SCI.

## Conclusion

In the present trial, no safety concerns were identified, and Muse cell transplantation demonstrated good tolerability. Future clinical trials with appropriate study designs incorporating a control arm will clarify the definitive efficacy of single-dose allogenic Muse cell treatment with intravenous administration to treat SCI.

## Data Availability

The datasets used and/or analyzed during the current study are available from the corresponding author on reasonable request.
