# Correction: Safety and feasibility of intravenous administration of a single dose of allogenic-Muse cells to treat human cervical traumatic spinal cord injury: a clinical trial

**DOI:** 10.1186/s13287-024-04044-0

**Published:** 2024-11-06

**Authors:** Masao Koda, Shiro Imagama, Hiroaki Nakashima, Sadayuki Ito, Naoki Segi, Jun Ouchida, Kota Suda, Satoko Harmon Matsumoto, Miki Komatsu, Toshiki Endo, Shinsuke Suzuki, Satoshi Inami, Haruki Ueda, Masayuki Miyagi, Gen Inoue, Masashi Takaso, Keiji Nagata, Hiroshi Yamada, Naosuke Kamei, Toshio Nakamae, Hidenori Suzuki, Norihiro Nishida, Masahiro Funaba, Gentaro Kumagai, Takeo Furuya, Yu Yamato, Toru Funayama, Hiroshi Takahashi, Masashi Yamazaki

**Affiliations:** 1 Department of Orthopedic Surgery, Faculty of Medicine, University of Tsukuba, Tsukuba, Japan; 2 Department of Orthopaedics/Rheumatology/Hand Surgery, Nagoya University Graduate School of Medicine, Nagoya, Japan; 3 Hokkaido Spinal Cord Injury Center, Bibai, Japan; 4 Department of Neurosurgery, Sendai Medical Center, Sendai, Japan; 5 Division of Neurosurgery, Tohoku Medical and Pharmaceutical University, Sendai, Japan; 6 Department of Orthopedic Surgery, Dokkyo Medical University, Mibu, Japan; 7 Department of Orthopedic Surgery, Kitasato University School of Medicine, Sagamihara, Japan; 8 Orthopedic Surgery, Wakayama Medical University, Wakayama, Japan; 9 Department of Orthopaedic Surgery, Graduate School of Biomedical and Health Sciences, Hiroshima University, Hiroshima, Japan; 10 Department of Orthopedic Surgery, Yamaguchi University School of Medicine, Yamaguchi, Japan; 11 Department of Orthopaedic Surgery, Hirosaki University Graduate School of Medicine, Hirosaki, Japan; 12 Department of Orthopedic Surgery, Graduate School of Medicine, Chiba University, Chiba, Japan; 13 Department of Orthopedic Surgery, Hamamatsu University School of Medicine, Hamamatsu, Japan

**Correction: Stem Cell Research & Therapy (2024) 15:259**

10.1186/s13287-024-03842-w

The original article contains two errors which the authors wish to address:

1. On line 3 of page 3, the sentence should simply read, ‘B1 and B2, which indicates complete […]’, and the word ‘(Ref)’ should be disregarded.
2. In Fig. 2C, the Y-axis label should instead read as ‘Change in total motor score’, and the word ‘lower’ should be disregarded’.

